# Clinical Features and Immunological Markers Associated with Invasive Pulmonary Fungal Disease in Organizing Pneumonia: A Matched Case–Control Study

**DOI:** 10.3390/jof12100716

**Published:** 2026-09-23

**Authors:** Longzhen Lu, Yun Li, Zhiwei Long, Lejin Liang, Xidong Wang, Hongling Liu, Juan Cao, Shaoqiang Li, Yan Wang, Zhengtu Li, Feng Ye

**Affiliations:** 1 National Clinical Research Center for Respiratory Disease, State Key Laboratory of Respiratory Disease, National Center for Respiratory Medicine, Guangzhou Institute of Respiratory Health, The First Affiliated Hospital of Guangzhou Medical University, Guangzhou 510145, China; 15007882632@163.com (L.L.); liyun.welcom@163.com (Y.L.); bobolong2023@163.com (Z.L.); lejinn@163.com (L.L.); wangxidong3@163.com (X.W.); 13640989193@163.com (H.L.); juanziapple@126.com (J.C.); lishaoqiang86@163.com (S.L.); 13535263176@163.com (Y.W.); 2 The Second School of Clinical Medicine, Guangzhou Medical University, Guangzhou 510180, China

**Keywords:** organizing pneumonia, invasive pulmonary fungal disease, immunological markers, case–control study, T-lymphocyte subsets

## Abstract

Background/Objectives: Invasive pulmonary fungal disease (IPFD) is a serious complication of organizing pneumonia (OP), but associated clinical and immunological features remain poorly defined. Methods: We conducted a retrospective 1:2 age- and sex-matched case–control study of 126 adults with OP, including 42 with concomitant IPFD and 84 controls. Clinical, radiological, immunosuppressive exposure and admission lymphocyte-subset data were analyzed using a full-cohort clinical model and an immunological subgroup model (*n* = 39). Results: Pre-infection glucocorticoid and immunosuppressant exposure did not differ between groups. Invasive mechanical ventilation was more frequent with IPFD (59.5% vs. 21.4%, *p* < 0.001). Pleural effusion was associated with higher odds of IPFD (OR = 3.263, 95% CI: 1.237–8.607), whereas coexisting interstitial lung disease was associated with lower odds (OR = 0.168, 95% CI: 0.066–0.426). In the immunological subgroup, a lower CD4^+^ T-cell percentage was associated with IPFD (OR = 0.914, 95% CI: 0.849–0.983, *p* = 0.015), although statistical significance was lost after exclusion of possible IPFD cases. Conclusions: Pleural effusion and lower CD4^+^ T-cell percentage were associated with IPFD in OP; the potential clinical utility of lymphocyte-subset assessment should be evaluated prospectively.

## 1. Introduction

Organizing pneumonia (OP) is a clinicopathological syndrome characterized by the formation of granulation tissue within alveolar spaces and terminal bronchioles [1,2]. Glucocorticoids are widely regarded as the first-line treatment for OP [3,4]. However, prolonged high-dose glucocorticoid therapy exerts a dual effect: while suppressing excessive inflammatory responses, it substantially increases the risk of opportunistic infections, among which invasive pulmonary fungal disease (IPFD) is of particular concern [5,6,7].

In clinical practice, OP and IPFD share considerable overlap in both symptoms and high-resolution computed tomography (HRCT) findings, as both may present with patchy consolidation, ground-glass opacities, nodules and migratory lesions [8,9]. Cordier [3] noted that the radiological manifestations of OP are highly heterogeneous and difficult to distinguish from various infectious diseases. Drakopanagiotakis et al. [4] compared cryptogenic organizing pneumonia (COP) with secondary organizing pneumonia (SOP) and found that their imaging features were similar, with both requiring differentiation from infectious aetiologies. Notably, the relationship between OP and IPFD is bidirectional: on the one hand, patients with OP may develop secondary fungal infections during glucocorticoid therapy [10,11]; on the other hand, fungal infections themselves can trigger OP-like immune responses [12,13]. This complex pathophysiological interplay makes it difficult for clinicians to establish the causal sequence when OP and IPFD coexist, posing a considerable diagnostic and therapeutic challenge.

Host defense against fungi is critically dependent on the integrity of cell-mediated immunity, particularly CD4^+^ T-cell-mediated effector mechanisms [14,15,16]. Espinosa et al. [14] systematically described the central role of cytokines in regulating the differentiation of fungus-specific CD4^+^ T cells. Previous studies have demonstrated that immunosuppressant-induced imbalance and depletion of T-lymphocyte subsets constitute important host factors for opportunistic fungal infections [15,17,18]. Flesch et al. [19] showed that IFN-γ-activated macrophages possess cryptococcal killing capacity, while Thakur et al. [20] revealed the critical role of effector T-helper cells in invasive aspergillosis. More recent human studies have demonstrated heterogeneous immune phenotypes in invasive mould disease and antifungal activity of unconventional T-cell subsets against A. fumigatus, further illustrating the complexity of antifungal cellular immunity [21,22]. Nevertheless, existing studies on IPFD complicating OP have largely been limited to small case series [11,13], lacking matched cohort designs with rigorous control of confounders. Moreover, most have focused on macroscopic clinical features without exploring the immunological mechanisms underlying fungal susceptibility in patients with comparable glucocorticoid exposure.

Against this background, we conducted a 1:2 matched case–control study retrospectively analyzing clinical data from 126 OP patients at our centre. The aim was to characterize clinical and radiological features associated with IPFD in OP while accounting for iatrogenic immunosuppressive exposure, and to explore potential immunological markers using peripheral blood lymphocyte-subset profiles at admission.

## 2. Methods

### 2.1. Study Design and Participants

This single-centre, retrospective, matched case–control study was conducted at the First Affiliated Hospital of Guangzhou Medical University (National Center for Respiratory Medicine), China. The study protocol was approved by the institutional ethics committee (approval numbers ES-2024-177-01 and ES-2024-177-02, date of approval: 9 October 2024), and the requirement for individual informed consent was waived given the retrospective nature of the study.

We screened consecutive adult patients (aged ≥18 years) diagnosed with OP at our institution between December 2012 and September 2024. Patients were required to have complete clinical, radiological and laboratory or pathological records. We excluded patients with active pulmonary tuberculosis or HIV infection, those with pre-existing conditions causing severe immunodeficiency unrelated to OP management (e.g., hematological malignancies undergoing chemoradiotherapy), and duplicate admissions (only the index episode was retained).

Using a 1:2 matched case–control design [23], we assigned eligible OP patients with concomitant IPFD to the fungal group and matched each case to two contemporaneous OP patients without evidence of fungal infection (control group) by age (±3 years) and sex. Controls had no evidence of IPFD based on the available clinical and radiological assessment and, when clinically indicated, microbiological testing during the corresponding hospitalization; fungal investigations were not performed according to a uniform study protocol. This yielded a final cohort of 126 patients (42 cases and 84 controls).

### 2.2. Definitions

OP was diagnosed in accordance with the ATS/ERS classification of idiopathic interstitial pneumonias [1,24], based on compatible clinical features and characteristic HRCT findings, with histopathological confirmation by lung biopsy when available. In the absence of biopsy, the diagnosis was established on the basis of compatible clinicoradiological features and multidisciplinary discussion [3,25]. Of the 126 patients, 37 (29.4%) had histopathological confirmation of OP, whereas the remaining 89 (70.6%) were diagnosed on compatible clinicoradiological grounds and confirmed through multidisciplinary discussion.

For study purposes, IPFD cases were retrospectively classified according to the 2020 revised EORTC/MSGERC consensus definitions [26] and the Chinese national guidelines for invasive pulmonary fungal disease (2025 edition) [27]. Cases were categorized as proven, probable or possible according to the applicable host, clinical/radiological and mycological criteria. Possible cases did not fulfil the study criteria required for classification as probable or proven IPFD. For each IPFD case, the specimen source and recorded diagnostic evidence were additionally abstracted from the medical record, including histopathology, bronchoalveolar lavage fluid (BALF) fungal culture, galactomannan (GM), cryptococcal antigen (CrAg), 1,3-β-D-glucan (BDG), and BALF metagenomic next-generation sequencing (mNGS), when available. Species-level *Aspergillus* identification was recorded when documented; otherwise, cases were reported as *Aspergillus* spp. An isolated positive *Aspergillus* result from a non-sterile respiratory specimen was not considered sufficient on its own to establish invasive disease; *Aspergillus* findings were interpreted together with the applicable host, clinical/radiological and mycological criteria to reduce misclassification of colonization.

### 2.3. Data Collection

Clinical data were extracted from the electronic medical record system and included: demographics and comorbidities (age, sex, smoking history and coexisting ILD); pre-infection immunosuppressive exposure (systemic glucocorticoid use—initial peak dose, cumulative dose and total duration, all expressed as prednisone equivalents [28]—and prior immunosuppressant therapy); presenting symptoms and HRCT findings (consolidation, ground-glass opacities, pleural effusion, cavitation and the air-crescent sign); laboratory parameters (complete blood count, C-reactive protein and procalcitonin) and peripheral blood lymphocyte subsets determined by standardized flow cytometry at admission (CD4^+^ T-helper-cell and CD8^+^ T-cell percentages); and microbiological results (fungal culture, histopathology and molecular assays).

The primary outcome was identification of factors independently associated with IPFD in patients with OP. Secondary outcomes comprised in-hospital markers of disease severity, including invasive mechanical ventilation, ICU admission and in-hospital all-cause mortality. These variables were treated as clinical outcomes rather than candidate predictors because their temporal relationship to IPFD could not be established consistently from the retrospective records.

### 2.4. Statistical Analysis

Analyses were performed using IBM SPSS Statistics for Windows, version 25.0 (IBM Corp., Armonk, NY, USA). Continuous variables are presented as mean ± SD or median (interquartile range) depending on distribution and were compared using the independent-samples *t*-test or Mann–Whitney *U* test, respectively. Categorical variables are reported as *n* (%) and were compared using the Pearson chi-squared test when its assumptions were satisfied and Fisher’s exact test otherwise.

Multivariable logistic regression was performed using a two-model strategy. Model I (full cohort, *n* = 126) included clinical variables with *p* < 0.10 in univariable analysis, using Pearson chi-squared *p* values for categorical variables when its assumptions were satisfied; this yielded coexisting ILD, pleural effusion and hypoalbuminaemia. The *p* < 0.10 threshold was used pragmatically to limit model complexity relative to the available events. Given the limited subgroup size and interdependence among lymphocyte-subset measures, Model II was intentionally kept parsimonious and included ILD and CD4^+^ T-cell percentage in patients with complete lymphocyte-subset data (*n* = 39; 21 cases and 18 controls); CD4^+^ T-cell percentage showed the strongest univariable association among the lymphocyte-subset measures. Model I contained 42 IPFD events and three covariates (14 events per variable), and Model II contained 21 events and two covariates (10.5 events per variable). Multicollinearity was assessed using variance inflation factors, and no interaction terms were fitted because of the limited event counts. Model calibration was assessed using the Hosmer–Lemeshow test. Additional immunological variables with univariable *p* < 0.10 were examined separately in exploratory ILD-adjusted models. An exploratory ROC analysis was used to identify the CD4^+^ T-cell percentage cutoff maximizing the Youden index, which was subsequently evaluated in an ILD-adjusted logistic regression model. AUCs are reported with 95% confidence intervals estimated using the DeLong method. Because controls were individually matched to cases by age and sex, the robustness of Model I was additionally assessed using conditional logistic regression stratified by matched set. A corresponding conditional analysis was not performed for Model II because complete-case restriction for lymphocyte-subset data left too few informative matched sets to support a stable matched-set model.

Diagnostic robustness was further evaluated in two steps. First, a subgroup consistency analysis compared proven/probable (*n* = 31) with possible (*n* = 11) cases within the fungal group. Second, a sensitivity analysis was conducted after excluding possible cases together with their matched controls (purified cohort: *n* = 93, 31 cases and 62 controls; immunological subgroup: *n* = 27), and the logistic regression models were reconstructed. All tests were two-sided, with *p* < 0.05 considered statistically significant.

## 3. Results

### 3.1. Baseline Characteristics and Clinical Outcomes

After 1:2 matching by age and sex, 126 patients were included (42 in the fungal group and 84 controls). As expected from the matching procedure, the groups were well balanced for age and sex; smoking history also did not differ significantly. Coexisting ILD, however, was substantially more prevalent in the control group than in the fungal group (86.9% vs. 52.4%, *p* < 0.001). Pre-infection systemic glucocorticoid exposure—including the proportion of users, cumulative dose and duration—and prior immunosuppressant use were comparable between groups (all *p* > 0.05) (Table 1). Regarding disease severity, the fungal group had a markedly higher rate of invasive mechanical ventilation than the OP-alone group (59.5% vs. 21.4%, *p* < 0.001), whereas ICU admission rates did not differ significantly. No in-hospital deaths occurred in either group (Table 2).

### 3.2. Pathogen Distribution and Diagnostic Classification in the Fungal Group

Among the 42 IPFD cases, 12 (28.6%) were classified as proven, 19 (45.2%) as probable and 11 (26.2%) as possible. Recorded pathogen attribution included *Aspergillus* species in 16 cases (38.1%) and *Cryptococcus* species in 14 (33.3%), together representing the predominant pathogens (71.4%, 30/42). *Pneumocystis jirovecii* was identified in 2 cases (4.8%), mixed infections involving two or more pathogens in 7 (16.7%), and the causative organism remained unspecified in 3 (7.1%) (Table 3). Case-level pathogen assignment, diagnostic category, specimen source and recorded diagnostic evidence are summarized in Supplementary Table S5. Among the 21 cases with *Aspergillus* involvement, including mixed fungal infections, A. fumigatus was documented in four cases; the remaining 17 cases were identified only to the genus level and are reported as *Aspergillus* spp.

### 3.3. Clinical and Radiological Features

The two groups did not differ in the frequency of fever, cough, sputum production or dyspnoea (all *p* > 0.05). Chest HRCT findings showed substantial overlap, with consolidation, ground-glass opacities, nodules and the air-bronchogram sign similarly distributed between groups. Pleural effusion was more frequent in the fungal group than in controls (33.3% vs. 19.0%; χ^2^ = 3.150, *p* = 0.076), meeting the *p* < 0.10 screening criterion for Model I. The air-crescent sign was observed exclusively in the fungal group (2/42 [4.8%] vs. 0/84; Fisher’s exact *p* = 0.109); because it did not meet the Model I entry criterion and occurred in only two patients, it was retained as a descriptive radiological finding rather than entered into multivariable regression. Both patients with an air-crescent sign had pulmonary aspergillosis (Table 4).

### 3.4. Laboratory and Immunological Findings

Routine inflammatory markers—white blood cell count, albumin, C-reactive protein and procalcitonin—and cytokine levels (IL-2, IL-4 and IL-6) were comparable between groups (all *p* > 0.05).

To assess potential selection bias related to incomplete lymphocyte-subset data, we compared patients with available immunological data (*n* = 39) with those without (*n* = 87). Apart from a younger age among patients with available immunological data, measured ILD prevalence, initial glucocorticoid dose, mechanical ventilation and ICU admission did not differ significantly (Supplementary Table S1). Nevertheless, because lymphocyte-subset testing was not performed uniformly and complete data were available for only 39 of 126 patients, residual selection bias cannot be excluded.

Among patients with available lymphocyte subset data (fungal group 21, controls 18), the CD4^+^ T-helper cell percentage was significantly lower in the fungal group (33.00% vs. 44.35%, *p* = 0.015), as was the CD4^+^/CD8^+^ ratio (1.12 vs. 1.79, *p* = 0.044). In contrast, absolute CD4^+^ and CD8^+^ T-cell counts did not differ between groups (both *p* > 0.05). IFN-γ was numerically higher in the fungal group (2.05 vs. 1.19 pg/mL, *p* = 0.055) (Table 5, Figure 1). Additional immunological variables with univariable *p* < 0.10 were evaluated separately in exploratory ILD-adjusted models; none showed a statistically significant independent association with IPFD (Supplementary Table S4).

### 3.5. Multivariable Associations and Exploratory Modelling

In Model I (full-cohort clinical model, *n* = 126), pleural effusion was positively associated with IPFD (OR = 3.263, 95% CI: 1.237–8.607, *p* = 0.017), whereas coexisting ILD was inversely associated with IPFD (OR = 0.168, 95% CI: 0.066–0.426, *p* < 0.001). Hypoalbuminaemia, although included in Model I based on the univariable screening criterion, was not independently associated with IPFD after adjustment (OR = 0.284, 95% CI: 0.056–1.440, *p* = 0.129). In a matched-set conditional logistic sensitivity analysis, the principal Model I associations were materially unchanged: pleural effusion remained associated with higher odds of IPFD (OR = 3.478, 95% CI: 1.149–10.530, *p* = 0.027), whereas coexisting ILD remained inversely associated with IPFD (OR = 0.131, 95% CI: 0.039–0.436, *p* = 0.001); hypoalbuminaemia remained non-significant (OR = 0.412, 95% CI: 0.082–2.069, *p* = 0.282). Coexisting ILD was also inversely associated with IPFD in Model II (OR = 0.134, 95% CI: 0.021–0.868, *p* = 0.035). In the primary Model II (*n* = 39; 21 cases and 18 controls), higher CD4^+^ T-cell percentage was inversely associated with IPFD (OR = 0.914 per 1-percentage-point increase, 95% CI: 0.849–0.983, *p* = 0.015). No substantial multicollinearity was detected (all VIFs ≤ 1.078), and Hosmer–Lemeshow testing showed no evidence of gross lack of fit for either model (both *p* > 0.05). For clinical interpretability, exploratory ROC analysis identified 39.8% as the Youden-optimal CD4^+^ T-cell percentage cutoff (sensitivity, 81.0%; specificity, 61.1%). After adjustment for coexisting ILD, a CD4^+^ T-cell percentage ≤ 39.8% was associated with higher odds of IPFD (OR = 6.792, 95% CI: 1.433–32.199, *p* = 0.016) (Figure 2).

Exploratory ROC analyses were used to summarize the apparent discrimination of each model within its respective analysis population. Model I yielded an AUC of 0.744 (95% CI: 0.656–0.833) in the full cohort (*n* = 126), whereas Model II yielded an AUC of 0.812 (95% CI: 0.678–0.947) in the immunological subgroup (*n* = 39). Because the two models were evaluated in different analysis populations, their AUCs were not directly compared (Figure 3).

### 3.6. Sensitivity Analysis

A subgroup consistency analysis within the fungal group revealed no significant differences between proven/probable (*n* = 31) and possible (*n* = 11) cases in age, sex, mechanical ventilation rate (54.8% vs. 72.7%, *p* = 0.477), pleural effusion rate (32.3% vs. 36.4%, *p* = 1.000) or CD4^+^ T-cell percentage (32.8% vs. 33.0%, *p* = 0.719) (Supplementary Table S2).

We then excluded the 11 possible IPFD cases together with their matched controls and reconstructed the logistic regression models in the purified cohort (*n* = 93; 31 cases, 62 controls). The inverse association between coexisting ILD and IPFD remained similar to that in the primary analysis (OR = 0.167, 95% CI: 0.057–0.488, *p* = 0.001 vs. OR = 0.168). In the immunological subgroup (*n* = 27), the point estimate for CD4^+^ T-cell percentage remained directionally consistent with the primary analysis (OR = 0.922, 95% CI: 0.844–1.008), but the association no longer reached conventional statistical significance (*p* = 0.074) (Supplementary Table S3).

## 4. Discussion

In this matched case–control study, three principal observations emerged. First, patients with coexisting IPFD more frequently required invasive mechanical ventilation, indicating greater in-hospital disease severity; however, the retrospective design does not allow us to determine whether mechanical ventilation preceded, coincided with or followed recognition of IPFD. Second, coexisting ILD was independently associated with lower odds of IPFD, an inverse association that should not be interpreted as evidence of biological protection. Third, a lower CD4^+^ T-cell percentage was associated with IPFD in the small immunological subgroup, although this association became statistically non-significant after possible IPFD cases were excluded. These findings should therefore be interpreted as clinical and immunological associations rather than evidence of causal risk factors.

The pathogen spectrum in our fungal group was dominated by *Aspergillus* species (38.1%) and *Cryptococcus* species (33.3%). Although the proportion of cryptococcosis was relatively high, pulmonary cryptococcosis is well documented in Guangdong: a recent cohort from Jiangmen included 118 non-AIDS patients with pulmonary cryptococcosis, while a six-year study from eastern Guangdong identified pneumonia in 42 of 170 patients with cryptococcosis [29,30]. Nevertheless, our single-centre tertiary-referral cohort was not designed to estimate population-level pathogen prevalence. The relatively high proportion of *Cryptococcus* in this study may therefore reflect a combination of regional epidemiology, referral patterns, diagnostic practices and study-population characteristics rather than any single environmental exposure.

The strong inverse association between coexisting ILD and IPFD (OR = 0.168) should be interpreted cautiously and not as evidence that ILD is biologically protective. The marked imbalance in ILD prevalence between cases and controls despite age- and sex-matching suggests that residual confounding and selection processes may contribute to this association. Several non-mutually exclusive explanations are possible. Patients with established ILD may differ from those with isolated OP in surveillance intensity, referral pathways and the likelihood of undergoing fungal investigations. These differences may also contribute to referral, diagnostic and indication bias in this retrospective cohort. In addition, OP occurring in the setting of underlying ILD may represent a clinically distinct phenotype from isolated or cryptogenic OP [4,25]. Finally, although measured glucocorticoid exposure was similar between groups, retrospective summary measures cannot fully capture potential differences in treatment timing, pulse therapy, concomitant immunosuppressants or treatment changes around the diagnostic episode. The present data cannot distinguish among these explanations. Accordingly, the inverse ILD association should be regarded as cohort-specific and hypothesis-generating rather than evidence of a protective biological effect.

At the clinical and radiological level, our findings confirm the extensive phenotypic overlap between OP and IPFD: routine symptoms and core HRCT features were largely indistinguishable, consistent with previous reports [3,4]. Pleural effusion, however, was independently associated with IPFD (OR = 3.263). This is pathophysiologically plausible. Classic OP is confined to the alveolar spaces and terminal bronchioles and rarely involves the pleura [3,25], whereas invasive aspergillosis is characterized by angioinvasion that can cause parenchymal necrosis and structural disruption [31,32], occasionally extending to the pleural surface in severe or extensive disease, although pleural effusion is not a hallmark radiological feature [33]. The air-crescent sign was observed in only two patients, both with pulmonary aspergillosis, consistent with parenchymal necrosis and cavity formation during the course of invasive fungal infection [34]. Taken together, these observations suggest that pleural effusion or an air-crescent sign in a patient with otherwise typical OP imaging may warrant targeted evaluation for fungal co-infection.

The immunological subgroup showed a lower CD4^+^ T-cell percentage in patients with IPFD, whereas absolute CD4^+^ counts were not significantly different. This pattern should not be interpreted as direct evidence of CD4^+^ T-cell depletion or impaired cellular immunity. Because lymphocyte percentages are compositional measures, a lower CD4^+^ proportion may reflect relative changes in other lymphocyte populations rather than an isolated reduction in the CD4^+^ compartment. The accompanying difference in the CD4^+^/CD8^+^ ratio and the borderline B-lymphocyte finding further support caution when attributing this association to a single immune-cell lineage. Recent human immune profiling in invasive mould infection has likewise demonstrated heterogeneous immune phenotypes potentially shaped by the underlying disease, immunosuppressive treatment and active infection [21]. Moreover, human Vγ9Vδ2 T cells have demonstrated direct antifungal activity against A. fumigatus and other filamentous fungi, underscoring that antifungal cellular immunity extends beyond conventional CD4^+^ T-cell measures [22]. In the present study, lymphocyte subsets were measured at admission, and we cannot determine whether the lower CD4^+^ percentage preceded IPFD or resulted from the infection or its associated inflammatory state. We therefore interpret CD4^+^ T-cell percentage as an associated immunological marker rather than a causal susceptibility factor.

Several limitations warrant emphasis. First, the single-centre retrospective design is susceptible to selection, diagnostic and verification bias; exposure timing and fungal work-up could not be fully standardized across cases and controls, precluding firm temporal inference. Second, 11 of 42 IPFD cases were classified as possible IPFD without mycological confirmation; after their exclusion, the CD4^+^ T-cell estimate remained directionally similar but lost statistical significance (OR = 0.922, 95% CI: 0.844–1.008, *p* = 0.074). Third, lymphocyte-subset data were available for only 39 of 126 patients, limiting precision, disrupting much of the original matched-set structure, and leaving potential selection bias for Model II and the internally derived CD4^+^ cutoff. Fourth, the marked ILD imbalance after age- and sex-matching raises the possibility of residual confounding. Finally, because most OP diagnoses were based on compatible clinicoradiological findings and multidisciplinary assessment rather than histopathology, overlap with infection-associated organizing changes cannot be excluded; OP etiology was not subclassified, and species-level Aspergillus identification was incomplete. Prospective multicentre studies with standardized mycological and immunological assessment are needed to validate these associations.

In conclusion, pleural effusion and a lower CD4^+^ T-cell percentage were associated with coexisting IPFD in this retrospective OP cohort, whereas coexisting ILD was associated with lower odds of IPFD but should not be interpreted as biologically protective. The CD4^+^ finding and the exploratory percentage cutoff require validation because they were derived from a small subgroup and were attenuated in sensitivity analysis. These results support heightened clinical and mycological vigilance for IPFD in patients with OP while underscoring the need for prospective studies with systematic immune profiling and standardized fungal diagnostics.

## Figures and Tables

**Figure 1:**
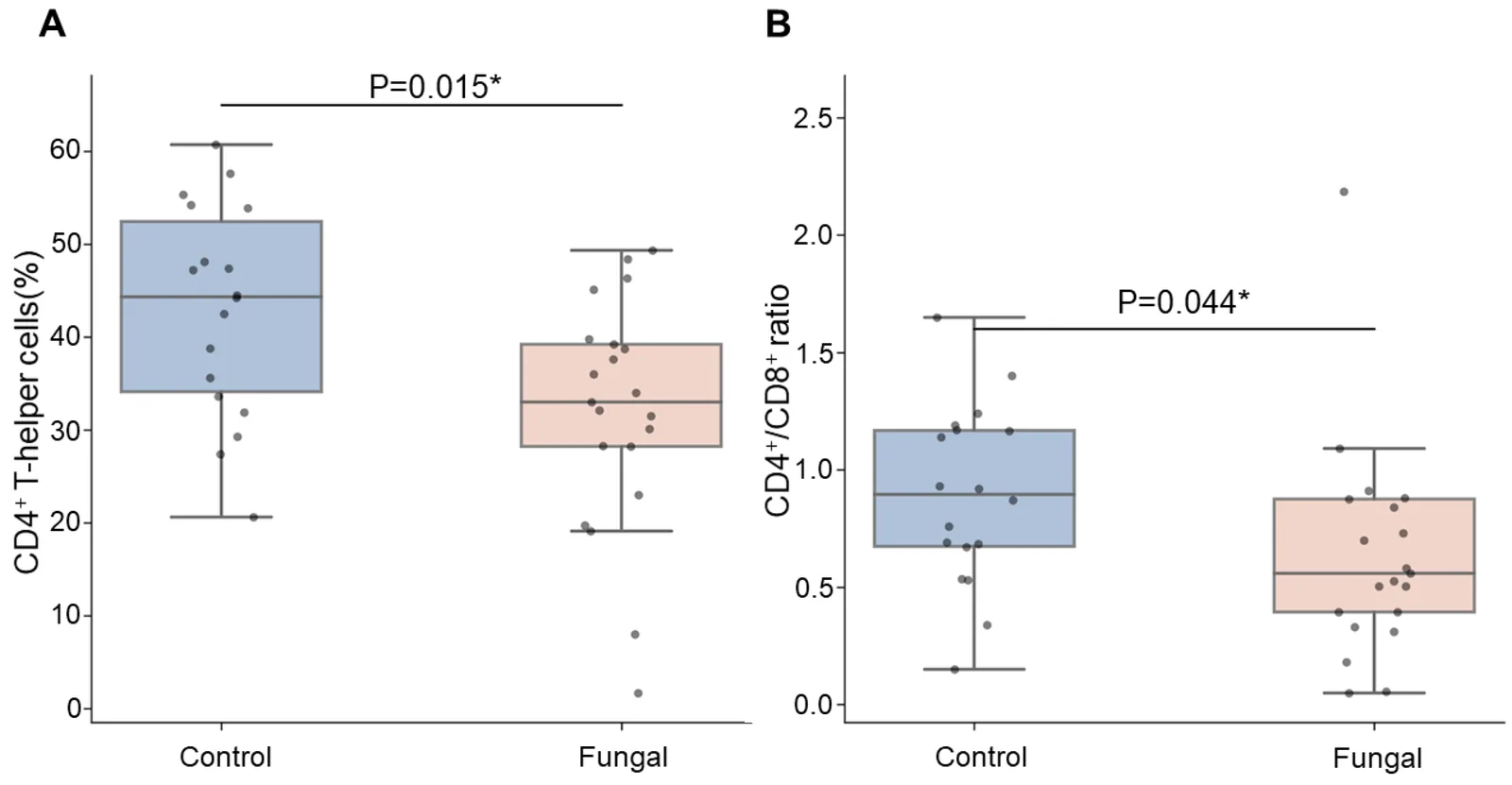
Peripheral blood CD4^+^ T-helper cell percentage (**A**) and CD4^+^/CD8^+^ ratio (**B**) in patients with organizing pneumonia. Data are shown for the fungal group (*n* = 21) and control group (*n* = 18). Box plots display the median (horizontal line), interquartile range (box), and whiskers extending to 1.5 times the interquartile range; individual points represent patient-level observations. * *p* < 0.05 (Mann–Whitney *U* test).

**Figure 2:**
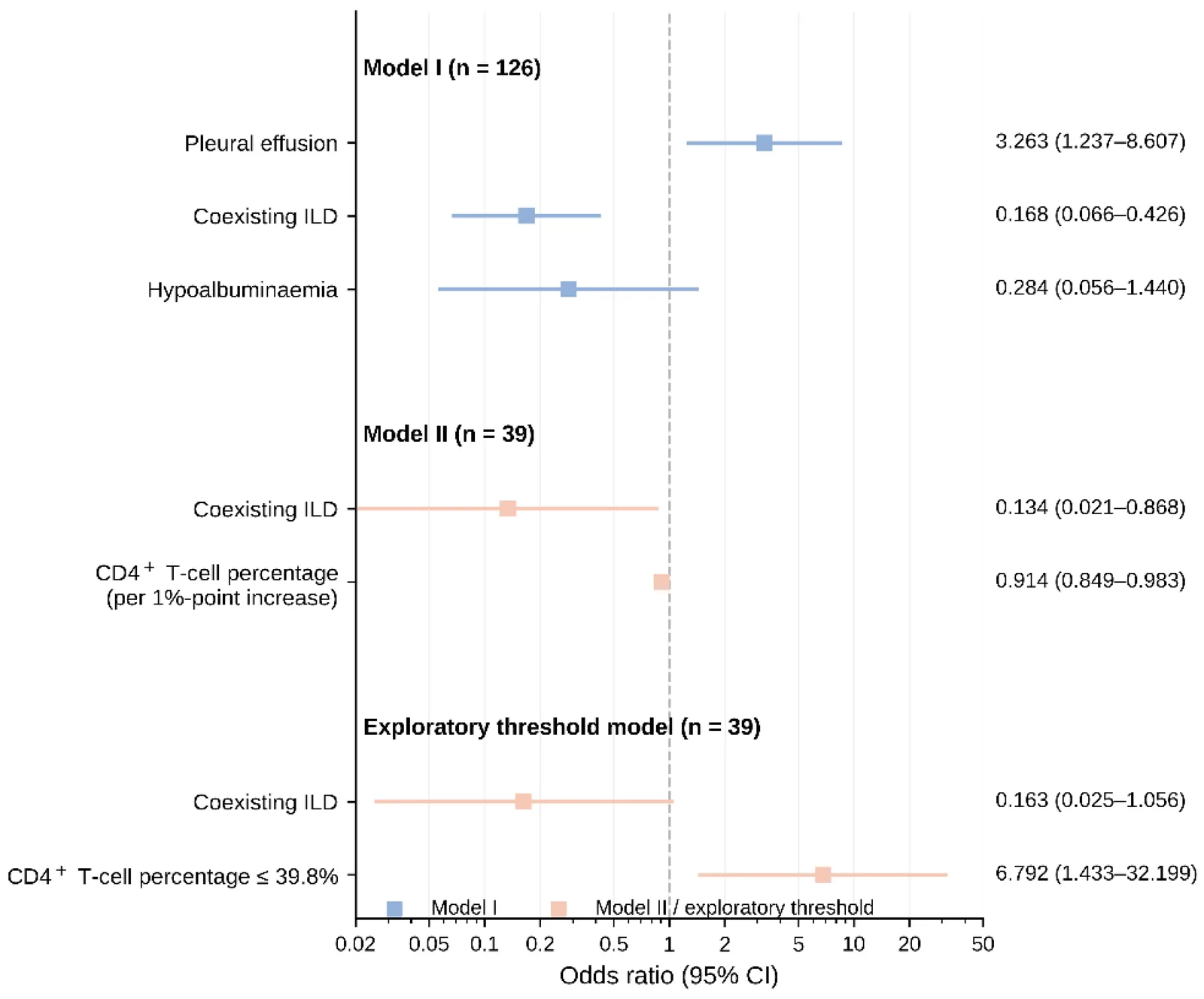
Forest plot of multivariable associations with invasive pulmonary fungal disease in patients with organizing pneumonia. Model I was fitted in the full cohort (*n* = 126) and included pleural effusion, coexisting interstitial lung disease (ILD) and hypoalbuminaemia. Model II was fitted in the immunological subgroup (*n* = 39) and included ILD and CD4^+^ T-cell percentage as a continuous variable. The exploratory threshold model shows the association of CD4^+^ T-cell percentage ≤ 39.8% after adjustment for ILD. Squares represent adjusted odds ratios (ORs), horizontal lines represent 95% confidence intervals (CIs), and the vertical dashed line indicates OR = 1.

**Figure 3:**
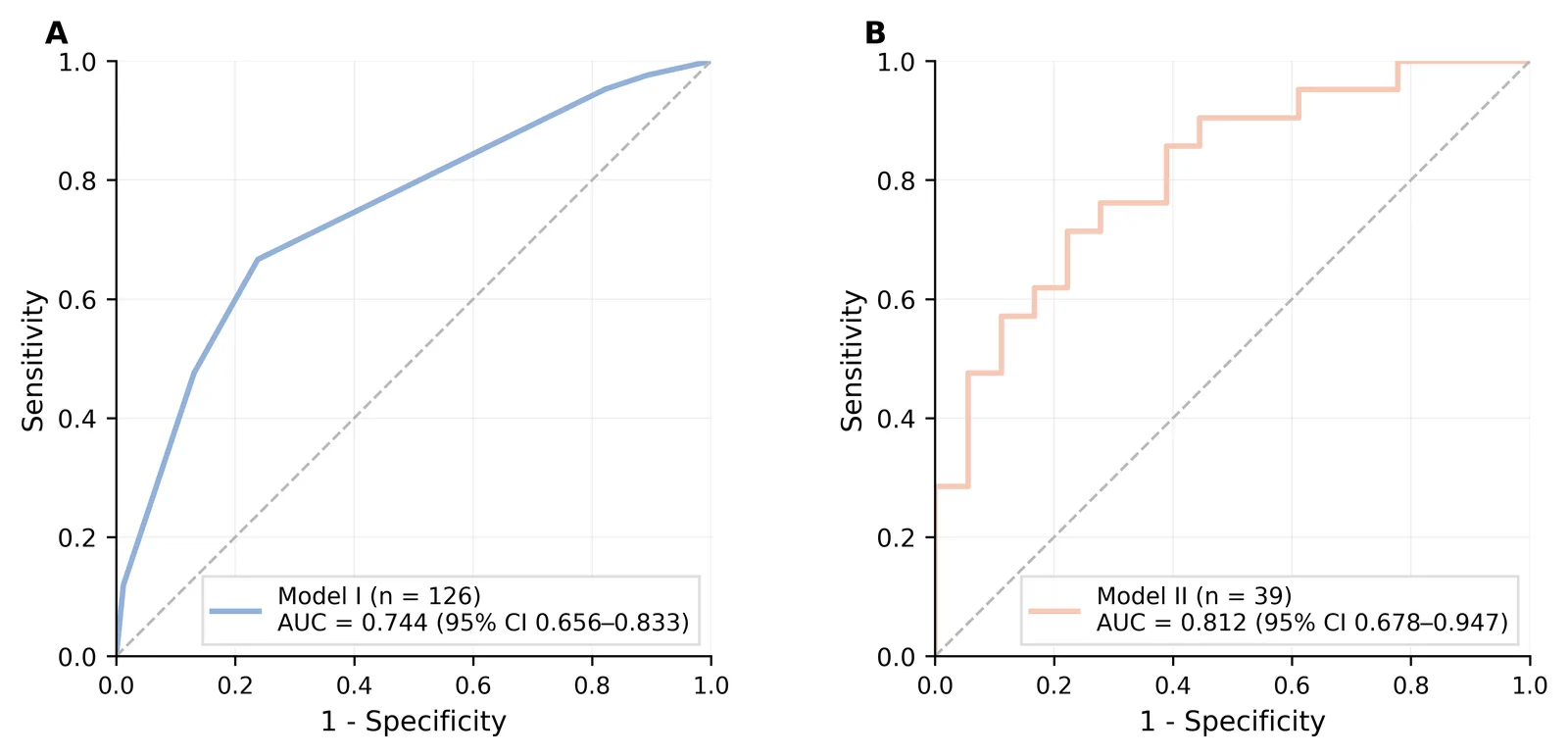
Receiver operating characteristic (ROC) curves summarizing the apparent discrimination of Model I in the full cohort ((**A**); *n* = 126) and Model II in the immunological subgroup ((**B**); *n* = 39). Areas under the curve (AUCs) are presented with 95% confidence intervals. The diagonal dashed line represents an AUC of 0.5. Because the models were estimated in different analysis populations, the AUCs were not directly compared.

**Table 1:** Baseline demographics, clinical characteristics and glucocorticoid exposure in patients with organizing pneumonia.

Characteristic	Fungal Group (*n* = 42)	Control Group (*n* = 84)	χ^2^/Z	*p* Value
Demographics				
Age, years, median (IQR)	60.0 (51.2, 67.0)	61.0 (51.0, 67.0)	0.041	0.967
Male, *n* (%)	34 (81.0)	68 (81.0)	0.000	1.000
Height, cm, median (IQR)	164.5 (161.2, 170.0)	165.0 (162.0, 170.0)	0.342	0.732
Weight, kg, median (IQR)	60.0 (52.8, 67.0)	62.0 (52.4, 68.5)	0.800	0.424
Smoking history, *n* (%)	19 (45.2)	31 (36.9)	0.812	0.367
Comorbidities, *n* (%)				
Interstitial lung disease	22 (52.4)	73 (86.9)	17.991	<0.001
Lung malignancy	6 (14.3)	7 (8.3)	— ^a^	0.356
Hypertension	5 (11.9)	18 (21.4)	1.702	0.192
Diabetes mellitus	4 (9.5)	12 (14.3)	0.573	0.449
COPD	4 (9.5)	8 (9.5)	— ^a^	1.000
Coronary artery disease	3 (7.1)	4 (4.8)	— ^a^	0.685
Bronchiectasis	2 (4.8)	2 (2.4)	— ^a^	0.600
Prior pulmonary tuberculosis	1 (2.4)	0 (0.0)	— ^a^	0.333
Hematological disease	1 (2.4)	2 (2.4)	— ^a^	1.000
Heart failure	0 (0.0)	2 (2.4)	— ^a^	0.552
Asthma	0 (0.0)	1 (1.2)	— ^a^	1.000
Stroke	0 (0.0)	1 (1.2)	— ^a^	1.000
Connective tissue disease	0 (0.0)	1 (1.2)	— ^a^	1.000
**Immunosuppressive exposure**				
Prior glucocorticoid use, *n* (%)	25 (59.5)	49 (58.3)	0.016	0.898
Prior immunosuppressant use, *n* (%)	27 (64.3)	53 (63.1)	0.017	0.896
Initial glucocorticoid dose, mg/d, median (IQR)	20.0 (0.0, 37.5)	10.0 (0.0, 40.0)	0.448	0.654
Cumulative glucocorticoid dose, mg, median (IQR)	140.0 (0.0, 607.5)	78.0 (0.0, 1218.0)	0.113	0.910
Duration of glucocorticoid therapy, d, median (IQR)	7.0 (0.0, 56.2)	4.0 (0.0, 105.5)	0.202	0.840
Lymphocyte count < 1.0 × 10^9^/L, *n* (%)	6 (14.3)	14 (16.7)	0.119	0.730
Hypoalbuminaemia (albumin < 30 g/L), *n* (%)	2 (4.8)	15 (17.9)	4.114	0.043

COPD, chronic obstructive pulmonary disease; IQR, interquartile range. Continuous variables were compared using the independent-samples *t*-test or Mann–Whitney *U* test, as appropriate. Categorical variables were compared using the Pearson chi-squared test when its assumptions were satisfied and Fisher’s exact test otherwise. All glucocorticoid doses are expressed as prednisone equivalents [28]. Fisher’s exact test was used; a indicates that χ^2^ value not applicable.

**Table 2:** Clinical outcomes in patients with organizing pneumonia.

Outcome	Fungal Group (*n* = 42)	Control Group (*n* = 84)	χ^2^	*p* Value
Invasive mechanical ventilation, *n* (%)	25 (59.5)	18 (21.4)	18.076	<0.001
ICU admission, *n* (%)	2 (4.8)	6 (7.1)	— ^a^	0.718
In-hospital mortality, *n* (%)	0 (0.0)	0 (0.0)	—	—

ICU, intensive care unit. Categorical outcomes were compared using the Pearson chi-squared test when its assumptions were satisfied and Fisher’s exact test otherwise. Fisher’s exact test was used; a indicates that χ^2^ value not applicable. No statistical comparison was performed for mortality as no deaths occurred in either group.

**Table 3:** Pathogen distribution and diagnostic classification in patients with organizing pneumonia complicated by invasive pulmonary fungal disease.

Pathogen	Proven (*n* = 12)	Probable (*n* = 19)	Possible (*n* = 11)	Total, *n* (%)
*Aspergillus* species	3	8	5	16 (38.1)
*Cryptococcus* species	8	3	3	14 (33.3)
*Pneumocystis jirovecii*	0	2	0	2 (4.8)
Mixed infection (≥2 pathogens)	1	6	0	7 (16.7)
Unspecified	0	0	3	3 (7.1)
Total, *n* (%)	12 (28.6)	19 (45.2)	11 (26.2)	42 (100.0)

Diagnostic classification was based on the 2020 revised EORTC/MSGERC consensus definitions [26] and the Chinese national guidelines for invasive pulmonary fungal disease (2025 edition) [27]. Percentages in the “Total” column represent the proportion of each pathogen among all IPFD cases (*n* = 42); percentages in the bottom row represent the proportion of each diagnostic category. For possible IPFD cases without pathogen-specific mycological confirmation, the pathogen category reflects the recorded clinical attribution and should not be interpreted as microbiologically confirmed identification.

**Table 4:** Clinical symptoms and high-resolution CT findings in patients with organizing pneumonia.

Feature	Fungal Group (*n* = 42)	Control Group (*n* = 84)	χ^2^/Z	*p* Value
**Clinical symptoms, *n* (%)**				
Cough	37 (88.1)	67 (79.8)	1.349	0.245
Sputum production	30 (71.4)	57 (67.9)	0.167	0.683
Fever	23 (54.8)	38 (45.2)	1.017	0.313
Dyspnoea	23 (54.8)	39 (46.4)	0.778	0.378
Weight loss	11 (26.2)	14 (16.7)	1.597	0.206
Haemoptysis	8 (19.0)	17 (20.2)	0.025	0.874
Chest pain	8 (19.0)	15 (17.9)	0.027	0.870
Chest tightness	7 (16.7)	13 (15.5)	0.030	0.863
Fatigue	6 (14.3)	12 (14.3)	0.000	1.000
Night sweats	1 (2.4)	3 (3.6)	— ^a^	1.000
**HRCT findings, *n* (%)**				
Patchy consolidation	37 (88.1)	73 (86.9)	0.036	0.850
Linear/reticular opacities	23 (54.8)	44 (52.4)	0.064	0.801
Nodules	19 (45.2)	37 (44.0)	0.016	0.899
Air-bronchogram sign	14 (33.3)	21 (25.0)	0.969	0.325
Pleural effusion	14 (33.3)	16 (19.0)	3.150	0.076
Ground-glass opacities	10 (23.8)	27 (32.1)	0.937	0.333
Air-crescent sign	2 (4.8)	0 (0.0)	— ^a^	0.109
Cavitation	2 (4.8)	2 (2.4)	— ^a^	0.600
Reversed halo sign	0 (0.0)	1 (1.2)	— ^a^	1.000
Halo sign	0 (0.0)	1 (1.2)	— ^a^	1.000

HRCT, high-resolution computed tomography. Categorical variables were compared using the Pearson chi-squared test when its assumptions were satisfied and Fisher’s exact test otherwise. Fisher’s exact test was used; a indicates that χ^2^ value not applicable.

**Table 5:** Laboratory parameters and lymphocyte subset analysis in patients with organizing pneumonia.

Parameter, Median (IQR)	Fungal Group (*n* = 42)	Control Group (*n* = 84)	Z	*p* Value
White blood cell count, ×10^9^/L	7.83 (6.49, 9.93)	8.50 (6.23, 10.80)	0.366	0.714
Neutrophil count, ×10^9^/L	5.12 (3.52, 7.35)	5.60 (3.80, 7.88)	0.132	0.895
Lymphocyte count, ×10^9^/L	1.42 (0.90, 2.00)	1.60 (1.20, 2.10)	1.299	0.194
Albumin, g/L	34.70 (31.80, 38.05)	35.20 (32.08, 38.52)	0.646	0.518
ESR, mm/h	45.50 (25.50, 77.75)	45.00 (18.00, 84.00)	0.399	0.690
CRP, mg/L	1.02 (0.24, 7.35)	1.05 (0.23, 8.90)	0.313	0.754
Procalcitonin, ng/mL	0.05 (0.05, 0.13)	0.05 (0.05, 0.17)	0.376	0.707
IL-2, pg/mL	1.26 (0.81, 1.54)	0.92 (0.46, 1.24)	1.559	0.119
IL-4, pg/mL	1.13 (0.51, 1.73)	1.23 (0.82, 1.83)	0.414	0.679
IL-6, pg/mL	6.72 (2.20, 17.20)	4.42 (3.03, 19.87)	0.135	0.893
IFN-γ, pg/mL	2.05 (1.35, 2.62)	1.19 (0.72, 2.03)	1.919	0.055
T lymphocytes, %	70.50 (59.50, 80.30)	71.90 (68.20, 81.40)	0.782	0.434
CD4^+^ T-helper cells, %	33.00 (28.20, 39.20)	44.35 (34.10, 52.45)	2.432	0.015
CD4^+^ T-helper cells, cells/μL	581.79 (196.73, 748.00)	594.40 (418.10, 918.00)	1.480	0.139
CD8^+^ T cells, %	26.90 (20.60, 35.50)	24.05 (21.75, 30.88)	0.451	0.652
CD8^+^ T cells, cells/μL	394.20 (265.74, 538.00)	430.60 (255.62, 729.40)	0.776	0.438
CD4^+^/CD8^+^ ratio	1.12 (0.79, 1.75)	1.79 (1.35, 2.34)	2.014	0.044
B lymphocytes, %	7.30 (4.00, 11.70)	10.80 (9.00, 14.00)	1.927	0.054
NK cells, %	14.70 (12.40, 24.00)	13.40 (8.90, 18.20)	1.526	0.127

ESR, erythrocyte sedimentation rate; CRP, C-reactive protein; IFN-γ, interferon-gamma; NK, natural killer; IQR, interquartile range. Lymphocyte subset data were available for 21 patients in the fungal group and 18 in the control group. Absolute CD4^+^ and CD8^+^ T-cell counts were derived from the absolute lymphocyte count and the corresponding subset proportion. All variables were non-normally distributed and compared using the Mann–Whitney *U* test.

## Data Availability

The datasets analyzed during the current study are not publicly available due to patient privacy considerations but are available from the corresponding author on reasonable request.

## Supplementary Materials

The following supporting information can be downloaded at: https://www.mdpi.com/article/10.3390/jof12100716/s1, Table S1. Baseline characteristics and clinical outcomes of patients with and without complete lymphocyte subset data. Table S2. Subgroup consistency analysis: comparison of proven/probable versus possible IPFD cases within the fungal group. Table S3. Sensitivity analysis: multivariable logistic regression after exclusion of possible IPFD cases. Table S4. Exploratory ILD-adjusted logistic regression analyses of additional immunological variables. Table S5. Case-level pathogen assignment, diagnostic classification, and supporting evidence for IPFD cases.

## References

[1] Oliveira D.S., Araújo Filho J.A., Paiva A.F.L., Ikari E.S., Chate R.C., Nomura C.H. (2018). Idiopathic interstitial pneumonias: Review of the latest American Thoracic Society/European Respiratory Society classification. Radiol. Bras..

[2] Cottin V., Cordier J.-F. (2012). Cryptogenic organizing pneumonia. Semin. Respir. Crit. Care Med..

[3] Cordier J.-F. (2006). Cryptogenic organising pneumonia. Eur. Respir. J..

[4] Drakopanagiotakis F., Paschalaki K., Abu-Hijleh M., Aswad B., Karagianidis N., Kastanakis E., Braman S.S., Polychronopoulos V. (2011). Cryptogenic and secondary organizing pneumonia: Clinical presentation, radiographic findings, treatment response, and prognosis. Chest.

[5] Lionakis M.S., Levitz S.M. (2018). Host Control of Fungal Infections: Lessons from Basic Studies and Human Cohorts. Annu. Rev. Immunol..

[6] Pappas P.G., Lionakis M.S., Arendrup M.C., Ostrosky-Zeichner L., Kullberg B.J. (2018). Invasive candidiasis. Nat. Rev. Dis. Primers.

[7] Lionakis M.S., Kontoyiannis D.P. (2003). Glucocorticoids and invasive fungal infections. Lancet.

[8] Faria I.M., Zanetti G., Barreto M.M., Rodrigues R.S., Araujo-Neto C.A., Silva J.L., Escuissato D.L., Souza A.S., Irion K.L., Mançano A.D. (2015). Organizing pneumonia: Chest HRCT findings. J. Bras. Pneumol..

[9] Zubovskaia A., Vazquez J.A. (2025). Invasive Aspergillosis in the Intensive Care Unit. J. Fungi.

[10] Lewis R.E., Kontoyiannis D.P. (2009). Invasive aspergillosis in glucocorticoid-treated patients. Med. Mycol..

[11] Wang Z., Wu C., Zhu R., Chen Z., Sun Z., Ma Q., Ji N., Huang M., Zhang M. (2020). Airway invasive aspergillosis with organizing pneumonia: A case report and review of literature. Ann. Transl. Med..

[12] Mukhopadhyay S., Gal A.A. (2010). Granulomatous lung disease: An approach to the differential diagnosis. Arch. Pathol. Lab. Med..

[13] Sakurai A., Yanai H., Ishida T., Kuwata H., Kamei K., Izumi S. (2017). Possible relationship between organizing pneumonia and chronic pulmonary aspergillosis: A case report and literature review. Respir. Investig..

[14] Espinosa V., Rivera A. (2012). Cytokines and the regulation of fungus-specific CD4 T cell differentiation. Cytokine.

[15] van de Veerdonk F.L., Netea M.G. (2010). T-cell Subsets and Antifungal Host Defenses. Curr. Fungal Infect. Rep..

[16] Romani L. (2011). Immunity to fungal infections. Nat. Rev. Immunol..

[17] Wüthrich M., Deepe G.S., Klein B. (2012). Adaptive immunity to fungi. Annu. Rev. Immunol..

[18] Brown G.D., Denning D.W., Gow N.A.R., Levitz S.M., Netea M.G., White T.C. (2012). Hidden killers: Human fungal infections. Sci. Transl. Med..

[19] Flesch I.E., Schwamberger G., Kaufmann S.H. (1989). Fungicidal activity of IFN-gamma-activated macrophages. Extracellular killing of Cryptococcus neoformans. J. Immunol..

[20] Thakur R., Anand R., Tiwari S., Singh A.P., Tiwary B.N., Shankar J. (2015). Cytokines induce effector T-helper cells during invasive aspergillosis; what we have learned about T-helper cells?. Front. Microbiol..

[21] Mellinghoff S.C., Thelen M., von Bergwelt-Baildon M., Schlößer H.A., Cornely O.A., Sprute R., Stemler J., Mayer L., Weskamm L.M., Friedrich M. (2025). Immune Phenotypes in Patients With Invasive Mould Infection Support the Use of PD-1 Inhibition as Potential Treatment Option. Mycoses.

[22] Koga S., Takazono T., Namie H., Okuno D., Ito Y., Nakada N., Hirayama T., Takeda K., Ide S., Iwanaga N. (2024). Human Vγ9Vδ2 T cells exhibit antifungal activity against Aspergillus fumigatus and other filamentous fungi. Microbiol. Spectr..

[23] Pearce N. (2016). Analysis of matched case-control studies. BMJ.

[24] Travis W.D., Costabel U., Hansell D.M., King T.E., Lynch D.A., Nicholson A.G., Ryerson C.J., Ryu J.H., Selman M., Wells A.U. (2013). An Official American Thoracic Society/European Respiratory Society Statement: Update of the International Multidisciplinary Classification of the Idiopathic Interstitial Pneumonias. Am. J. Respir. Crit. Care Med..

[25] King T.E., Lee J.S. (2022). Cryptogenic Organizing Pneumonia. N. Engl. J. Med..

[26] Donnelly J.P., Chen S.C., Kauffman C.A., Steinbach W.J., Baddley J.W., Verweij P.E., Clancy C.J., Wingard J.R., Lockhart S.R., Groll A.H. (2020). Revision and Update of the Consensus Definitions of Invasive Fungal Disease From the European Organization for Research and Treatment of Cancer and the Mycoses Study Group Education and Research Consortium. Clin. Infect. Dis..

[27] Chinese Thoracic Society (2025). Clinical practice guidelines for the diagnosis and management of invasive pulmonary fungal diseases (2025 Edition). Chin. J. Tuberc. Respir. Dis..

[28] Chinese Society of Endocrinology, China Endocrinology and Metabolism Specialist Alliance (2023). Clinical practice guidelines for glucocorticoid therapy (2023 edition). Chin. J. Endocrinol. Metab..

[29] Xiong P., Huang C., Zhong L., Huang L. (2023). Clinical and imaging characteristics of pulmonary cryptococcosis: A comparative analysis of 118 non-AIDS patients in China. Med. Mycol..

[30] Bilal H., Zhang D., Shafiq M., Khan M.N., Chen C., Khan S., Wang Q., Cai L., Awais M., Hu H. (2023). Cryptococcosis in Southern China: Insights from a Six-Year Retrospective Study in Eastern Guangdong. Infect. Drug Resist..

[31] Franquet T., Müller N.L., Giménez A., Guembe P., de La Torre J., Bagué S. (2001). Spectrum of pulmonary aspergillosis: Histologic, clinical, and radiologic findings. Radiographics.

[32] Stergiopoulou T., Meletiadis J., Roilides E., Kleiner D.E., Schaufele R., Roden M., Harrington S., Dad L., Segal B., Walsh T.J. (2007). Host-dependent patterns of tissue injury in invasive pulmonary aspergillosis. Am. J. Clin. Pathol..

[33] Kousha M., Tadi R., Soubani A.O. (2011). Pulmonary aspergillosis: A clinical review. Eur. Respir. Rev..

[34] Greene R.E., Schlamm H.T., Oestmann J.-W., Stark P., Durand C., Lortholary O., Wingard J.R., Herbrecht R., Ribaud P., Patterson T.F. (2007). Imaging findings in acute invasive pulmonary aspergillosis: Clinical significance of the halo sign. Clin. Infect. Dis..

