# Supplementary material for: Clinical Features and Immunological Markers Associated with Invasive Pulmonary Fungal Disease in Organizing Pneumonia: A Matched Case–Control Study
Source: J Fungi (Basel). 2026 Sep 23;12(10):716. doi: 10.3390/jof12100716 (PMC13705972; doi:10.3390/jof12100716)
Supplement: Supplementary file 1 [file jof-12-00716-s001.zip › jof-4372227-supplementary.pdf]

Supplementary Material

Table S1. Baseline characteristics and clinical outcomes of patients with and without complete lymphocyte subset data.

Characteristic	Full cohort (n = 126)	Complete data (n = 39)	Missing data (n = 87)	P value
**Baseline characteristics**				
Age, years, median (IQR)	61.0 (51.0, 67.0)	57.0 (47.0, 64.5)	62.0 (52.5, 69.5)	0.030
Male, n (%)	102 (81.0)	30 (76.9)	72 (82.8)	0.441
Interstitial lung disease, n (%)	95 (75.4)	28 (71.8)	67 (77.0)	0.530
Initial glucocorticoid dose, mg/d, median (IQR)	15.0 (0.0, 40.0)	20.0 (0.0, 40.0)	12.5 (0.0, 40.0)	0.170
**Clinical outcomes, n (%)**				
ICU admission	8 (6.3)	1 (2.6)	7 (8.0)	0.433[a]
Invasive mechanical ventilation	43 (34.1)	14 (35.9)	29 (33.3)	0.779

ICU, intensive care unit; IQR, interquartile range. Continuous variables were compared using the independent-samples *t*-test or Mann–Whitney *U* test as appropriate; categorical variables were compared using the Pearson chi-squared test when its assumptions were satisfied and Fisher's exact test otherwise. Glucocorticoid doses are expressed as prednisone equivalents. [a]Fisher's exact test was used.

Table S2. Subgroup consistency analysis: comparison of proven/probable versus possible IPFD cases within the fungal group.

Characteristic	Proven/Probable (n = 31)	Possible (n = 11)	Z	P value
**Baseline characteristics**				
Age, years, median (IQR)	61.0 (54.0, 66.5)	51.0 (46.0, 70.0)	0.587	0.557
Male, n (%)	26 (83.9)	8 (72.7)	—[a]	0.412
Interstitial lung disease, n (%)	16 (51.6)	6 (54.5)	—[a]	1.000
**Clinical outcomes and imaging, n (%)**				
Invasive mechanical ventilation	17 (54.8)	8 (72.7)	—[a]	0.477
Pleural effusion	10 (32.3)	4 (36.4)	—[a]	1.000
**Immunological parameter, median (IQR)**				
$CD4^+$ T-helper cells, %	32.8 (26.9, 38.8)	33.0 (32.1, 39.8)	0.360	0.719

IPFD, invasive pulmonary fungal disease; IQR, interquartile range. Continuous variables were compared using the Mann–Whitney *U* test. [a]Fisher's exact test was used; χ^2 value not applicable. $CD4^+$ T-cell percentage was compared only among IPFD cases with available lymphocyte-subset data; therefore, the denominators for this variable were smaller than the overall diagnostic-category totals shown in the column headings.

Table S3 Sensitivity analysis: multivariable logistic regression after exclusion of possible IPFD cases

Model	Variable	Adjusted OR	95% CI	P value
Model I (n = 93)	Pleural effusion	2.036	0.699–5.929	0.192
Model I (n = 93)	Coexisting ILD	0.167	0.057–0.488	0.001
Model I (n = 93)	Hypoalbuminaemia (albumin < 30 g/L)	0.431	0.083–2.239	0.317
Model II (n = 27)	Coexisting ILD	0.200	0.026–1.531	0.121
Model II (n = 27)	$CD4^+$ T-cell percentage	0.922	0.844–1.008	0.074

IPFD, invasive pulmonary fungal disease; ILD, interstitial lung disease; OR, odds ratio; CI, confidence interval. The purified cohort was obtained by excluding the 11 possible IPFD cases together with their 22 matched controls (Model I, n = 93); Model II was refitted in the corresponding immunological subgroup (n = 27). Adjusted ORs were estimated using the same covariate structure as the corresponding primary models.

Table S4 Exploratory ILD-adjusted logistic regression analyses of additional immunological variables

Variable	n	IPFD events	Univariable P value	Adjusted OR	95% CI	Adjusted P value
IFN-γ, pg/mL	42	19	0.055	1.738	0.924–3.272	0.087
$CD4^+/CD8^+$ ratio	39	21	0.044	0.672	0.332–1.361	0.270
B lymphocytes, %	38	21	0.054	0.904	0.808–1.012	0.079

IPFD, invasive pulmonary fungal disease; ILD, interstitial lung disease; OR, odds ratio; CI, confidence interval; IFN-γ, interferon-gamma. Each immunological variable was evaluated in a separate logistic regression model adjusted for coexisting ILD. ORs are expressed per 1-unit increase in the listed variable (1 pg/mL for IFN-γ, 1.0 unit for the $CD4^+/CD8^+$ ratio, and 1 percentage point for B lymphocytes). These analyses were exploratory and were not used to replace the primary Model II.

Table S5. Case-level pathogen assignment, diagnostic classification, and supporting evidence for IPFD cases

Study ID	Pathogen(s)	Diagnostic category	Specimen/source	Recorded diagnostic evidence	*Aspergillus* identification
IPFD-01	*Aspergillus* fumigatus	Proven	Tissue	Histopathological evidence of fungal infection	A. fumigatus
IPFD-02	*Aspergillus* fumigatus	Proven	Tissue	Histopathological evidence of fungal infection	A. fumigatus
IPFD-03	*Aspergillus* fumigatus	Proven	Tissue	Histopathological evidence of fungal infection	A. fumigatus

IPFD-04	*Aspergillus* spp.	Probable	BALF	Positive BALF fungal culture	*Aspergillus* spp. (species not identified)
IPFD-05	*Aspergillus* spp.	Probable	BALF	Positive BALF mNGS	*Aspergillus* spp. (species not identified)
Study ID	Pathogen(s)	Diagnostic category	Specimen/source	Recorded diagnostic evidence	*Aspergillus* identification
IPFD-06	*Aspergillus* spp.	Probable	BALF	Positive BALF fungal culture; Positive BALF mNGS	*Aspergillus* spp. (species not identified)
IPFD-07	*Aspergillus* spp.	Probable	Biomarker sample (source not specified)	GM positive; Compatible imaging findings	*Aspergillus* spp. (species not identified)
IPFD-08	*Aspergillus* spp.	Probable	Biomarker sample (source not specified)	GM positive; Compatible imaging findings	*Aspergillus* spp. (species not identified)
IPFD-09	*Aspergillus* spp.	Probable	BALF	Positive BALF mNGS	*Aspergillus* spp. (species not identified)
IPFD-10	*Aspergillus* spp.	Probable	BALF	Positive BALF fungal culture	*Aspergillus* spp. (species not identified)
IPFD-11	*Aspergillus* spp.	Probable	BALF	Positive BALF mNGS; Compatible imaging findings	*Aspergillus* spp. (species not identified)
IPFD-12	*Aspergillus* spp.	Possible	Imaging	Compatible imaging findings	*Aspergillus* spp. (species not identified)
IPFD-13	*Aspergillus* spp.	Possible	Biomarker sample (source not specified)	BDG positive	*Aspergillus* spp. (species not identified)
IPFD-14	*Aspergillus* spp.	Possible	Imaging	Compatible imaging findings	*Aspergillus* spp. (species not identified)
IPFD-15	*Aspergillus* spp.	Possible	Imaging	Compatible imaging findings	*Aspergillus* spp. (species not identified)
IPFD-16	*Aspergillus* spp.	Possible	Imaging	Compatible imaging findings	*Aspergillus* spp. (species not identified)
IPFD-17	*Cryptococcus* spp.	Proven	Tissue	Histopathological evidence of fungal infection	—
IPFD-18	*Cryptococcus* spp.	Proven	Tissue	Histopathological evidence of fungal infection	—
IPFD-19	*Cryptococcus* spp.	Proven	Tissue	Histopathological evidence of fungal infection	—

IPFD-20	*Cryptococcus* spp.	Proven	Tissue; BALF; Biomarker sample (source not specified)	Histopathological evidence of fungal infection; CrAg positive; Positive BALF mNGS	—
IPFD-21	*Cryptococcus* spp.	Proven	Tissue	Histopathological evidence of fungal infection	—
IPFD-22	*Cryptococcus* spp.	Proven	Tissue	Histopathological evidence of fungal infection; Compatible imaging findings	—
IPFD-23	*Cryptococcus* spp.	Proven	Tissue	Histopathological evidence of fungal infection	—
IPFD-24	*Cryptococcus* spp.	Proven	Tissue	Histopathological evidence of fungal infection	—
IPFD-25	*Cryptococcus* spp.	Probable	BALF; Biomarker sample (source not specified)	CrAg positive; Positive BALF mNGS	—
IPFD-26	*Cryptococcus* spp.	Probable	Biomarker sample (source not specified)	CrAg positive; Compatible imaging findings	—
IPFD-27	*Cryptococcus* spp.	Probable	Biomarker sample (source not specified)	CrAg positive	—
IPFD-28	*Cryptococcus* spp.	Possible	Imaging	Compatible imaging findings	—
IPFD-29	*Cryptococcus* spp.	Possible	Imaging	Compatible imaging findings	—
IPFD-30	*Cryptococcus* spp.	Possible	Imaging	Compatible imaging findings	—
IPFD-31	*Pneumocystis jirovecii*	Probable	BALF	BALF mNGS detected P. jirovecii; Compatible imaging findings	—
IPFD-32	*Pneumocystis jirovecii*	Probable	BALF	BALF mNGS detected P. jirovecii	—
IPFD-33	*Aspergillus* fumigatus + *Pneumocystis jirovecii*	Proven	Tissue; BALF; Biomarker sample (source not specified)	Histopathological evidence of fungal infection; GM positive; Positive BALF mNGS	A. fumigatus
IPFD-34	*Aspergillus* spp. + other fungal pathogen	Probable	BALF	Positive BALF mNGS	*Aspergillus* spp. (species not identified)
IPFD-35	*Cryptococcus* spp. + other	Probable	BALF	Positive BALF mNGS	—

	fungal pathogen				
IPFD-36	*Aspergillus* spp. + other fungal pathogen	Probable	BALF; Biomarker sample (source not specified)	GM positive; BDG positive; Positive BALF mNGS	*Aspergillus* spp. (species not identified)
IPFD-37	*Aspergillus* spp. + other fungal pathogen	Probable	BALF; Biomarker sample (source not specified)	GM positive; BDG positive; Positive BALF mNGS	*Aspergillus* spp. (species not identified)
IPFD-38	*Cryptococcus* spp. + other fungal pathogen	Probable	BALF; Biomarker sample (source not specified)	CrAg positive; Positive BALF mNGS	—
IPFD-39	*Aspergillus* spp. + other fungal pathogen	Probable	BALF	Positive BALF fungal culture; BALF mNGS	*Aspergillus* spp. (species not identified)
IPFD-40	Unspecified fungal pathogen	Possible	Imaging	Compatible imaging findings	—
IPFD-41	Unspecified fungal pathogen	Possible	Imaging	Compatible imaging findings	—
IPFD-42	Unspecified fungal pathogen	Possible	Imaging	Compatible imaging findings	—

IPFD, invasive pulmonary fungal disease; BALF, bronchoalveolar lavage fluid; GM, galactomannan; CrAg, cryptococcal antigen; BDG, 1,3-β-D-glucan; mNGS, metagenomic next-generation sequencing. Diagnostic categories were assigned according to the complete study classification framework. The diagnostic evidence listed here is presented as documented in the source records; final classification was based on the combination of applicable host, clinical/radiological, and mycological criteria rather than on any single test result. Cases classified as possible IPFD did not meet the study criteria for probable or proven IPFD. A. fumigatus is reported when documented; otherwise, Aspergillus spp. denotes genus-level identification only. Mixed infection denotes involvement of two or more fungal pathogens. For possible IPFD cases without pathogen-specific mycological confirmation, the pathogen category reflects the recorded clinical attribution in the source medical record and does not imply microbiological identification. Study IDs are anonymized; rows are ordered by pathogen category and then by diagnostic category for presentation only.
